# Large-scale microcarrier culture of HEK293T cells and Vero cells in single-use bioreactors

**DOI:** 10.1186/s13568-019-0794-5

**Published:** 2019-05-24

**Authors:** Jianjun Yang, Patrick Guertin, Guodong Jia, Zhongliang Lv, Hongyan Yang, Dianwen Ju

**Affiliations:** 1 School of Pharmacy, Fudan University, Shanghai, 201203 China; 2 Minhang Hospital, Fudan University, 170 Xinsong Road, Shanghai, 201199 China; 3 Fast Trak China Center, Life Sciences, GE Healthcare, Shanghai, 201203 China; 4 Fast Trak US Center, Life Sciences, GE Healthcare, Marlborough, 01752 USA; 5 OBiO Technology (Shanghai) Corp., Ltd., Shanghai, 201321 China

**Keywords:** Process scale-up, HEK293T cells, Vero cells, Microcarriers, Bead-to-bead transfer, Single-use Bioreactor

## Abstract

Gene therapy and viral vaccine are becoming attractive therapeutic options for the treatment of different malignant diseases. Viral vector productions are often using static culture vessels and small volume stainless steel bioreactors (SSB). However, the yield of each vessel can be relatively low and multiple vessels often need to be operated simultaneously. This significantly increases labor intensity, production costs, contamination risks, and limits its ability to be scaled up, thus, creating challenges to meet the quantities required once the gene therapy or viral vaccine medicine goes into clinical phases or to market. Single-use bioreactor combining with microcarrier provides a good option for viral vector and vaccine production. The goal of the present studies was to develop the microcarrier bead-to-bead expansion and transfer process for HEK293T cells and Vero cells and scale-up the cultures to 50–200 l single-use bioreactors. Following microcarrier bead-to-bead transfer, the peak cell concentration of HEK293T cells reached 1.5 × 10^6^ cells/ml in XDR-50 bioreactor, whereas Vero cells reached 3.1 × 10^6^ cells/ml and 3.3 × 10^6^ cells/ml in XDR-50 bioreactor and XDR-200 bioreactor, respectively. The average growth rates reached 0.61–0.68/day. The successful microcarrier-based scaleup of these two cell lines in single-use bioreactors demonstrates potential large-scale production capabilities of viral vaccine and vector for current and future vaccines and gene therapy.

## Introduction

Gene therapies and viral vaccines are becoming another research hotspot after antibody-based therapeutics, both being widely used for the treatments of a vast array of diseases, including genetic diseases, cancers, cardiovascular diseases, metabolic diseases, and infectious diseases, etc. (Buzhor et al. 2014; Draper and Heeney 2010; Wang and Gao 2014). Normal or therapeutic genes are transferred into target cells through a specific way in order to correct faulty genes or play a critical role in disease treatment. Commonly used gene transfer vehicles are viral vectors, which include lentivirus, adenovirus, adenovirus associated virus, retrovirus, etc. (Humphreys and Sebastian 2018; Strobel et al. 2019). Currently, thousands of gene therapy projects and viral vaccine projects are in Investigation New Drug phases or clinical trial phases. In these projects, the preparation of viruses through HEK293T cells and static multilayer flasks, may still meet the demand (Levine et al. 2017), but as the projects enter clinical phase III or get approval into the market, large-scale production of viruses could become a limiting factor in the industrialization of gene therapy and viral vaccine (van der Loo and Wright 2016). Therefore, the establishment of large-scale HEK293T cell culture process for virus production has become very urgent.

Viral vaccines have been widely used in the prevention of variety of diseases, including rabies, influenza and smallpox, et al. (Barrett et al. 2017). Vero cells as a cellular substrate are extensive employed in the production of viral vaccines. At present, many projects use multiple static systems to directly inoculate multiple dozens of liters of bioreactors at the same time for production. This is the scale-out approach, as opposed to scale-up. Scaling-out could greatly limit the large-scale production of viral vaccines (Gallo-Ramirez et al. 2015). In this process, microcarrier bead-to-bead transfer technology is a restrictive factor. Besides this, as regulations become more stringent, it is recommended that more single-use technologies, such as single-use bioreactors, γ-irradiated microcarriers, will be used in production process to reduce cross-contamination and prepare for pandemic diseases. Therefore, it is very urgent to establish microcarrier bead-to-bead transfer process of Vero cells and scaleup the culture process in large-scale single-use bioreactor.

Microcarriers are small spheres with an approximate diameter of 150–200 microns. The microcarriers provide attachment surface for adherent cells growth. Compared to multilayer flasks, microcarriers can significantly reduce process complexity and labor intensity even in large-scale culture process (Maartens et al. 2017). The product contact portion of single-use bioreactor is composed of a single-use sterile plastic bag assembly which is used in place of traditional stainless steel tank. This eliminates time-consuming clean in place (CIP), steam in place (SIP), and cleaning validation procedures, the infrastructure to support those operations and significant decreases cross-contamination risks. An additional benefit of rapid batch-to-batch changeover is that it can reduce response time to pandemic disease. There has been a tendency of using disposable bioreactors for seed expansion and recombinant protein or virus production (Eibl et al. 2010; Shukla and Gottschalk 2013).

In the work reported here, HEK293T cells, Vero cells and Cytodex-1 microcarrier cultures for seed expansions were studied in series of spinner flasks and WAVE25 bioreactors. The technology of microcarrier bead-to-bead transfer was also established, which is a critical procedure for industry scale-up and production. Then HEK293T cells and Vero cells large-scale cultivations were conducted in XDR-50 and XDR-200 bioreactors, respectively. The developed processes can be used for virus and vaccine related production.

## Materials and methods

### Cell lines, culture media, buffers and detach reagents

HEK293T cells was provided by Obio Technology (Shanghai) Corp., Ltd., and originally purchased from American Type Culture Collection (ATCC, Manassas, VA, CRL-3216). Vero cells was obtained from Cell Bank of Typical Culture Preservation Committee, Chinese Academy of Sciences, and originally obtained from ATCC (CCL-81).

Minimum Essential Medium (MEM, #SH30024.01), Medium 199 (M199, #SH30253.01), DMEM (#SH30243.01), Fetal Bovine Serum (FBS, #SH30084.04IR) were provided by HyClone. HEK293T and Vero cells were grown in above mentioned basal media supplemented with 10% FBS under a 5% CO_2_ and 37 °C incubator. Dulbecco’s phosphate-buffered saline (D-PBS) without calcium, magnesium (#SH30028.03), 0.25% Trypsin/EDTA (1×, #SH30042.01) and 2.5% Trypsin (10×, #SV30037.01) were provided by HyClone.

### Microcarriers

Cytodex-1 microcarrier (#17044803) and Cytodex-1 Gamma microcarrier (#17548702) were ordered from GE Healthcare. The hydration and sterilization process of Cytodex-1 were carried out according to manufacturer’s guide Microcarrier Cell Culture Principles and Methods. Dry Cytodex-1 microcarriers were added into D-PBS (50 ml/g Cytodex-1) at least 4 h for hydration, then washed once using the same volume of D-PBS, and then autoclaved at 121 °C with 30 min. Cytodex-1 microcarriers were used in all HEK293T cell culture processes and Vero cell culture processes in spinner flasks and XDR-50 bioreactor. Cytodex-1 Gamma microcarriers are Gamma-sterilized and ready-to-use for rapid culture start-up. The gamma sterilized microcarriers only require hydration and conditioning, not a heat sterilization process. In this study, Cytodex-1 Gamma microcarriers were applied in Vero cell culture process in XDR-200 bioreactor.

### Cell culture medium screening

In this study, media screening experiments for cell growth of HEK293T cells and Vero cells were conducted firstly. Three basal media and one FBS were employed, including MEM, M199, DMEM, and FBS. All the basal media were supplied with 10% FBS and divided into 3 groups, naming as (I) MEM + FBS, (II) M199 + FBS, (III) DMEM + FBS.

HEK293T cells were thawed to the three groups of media individually. After 2 passages adaption, the medium screening process of HEK293T cells was performed in total fifteen T25 flasks. Each T25 flask was inoculated at 2 × 10^4^ cells/cm^2^ with 5 ml medium, and 5 flasks for each group. After 4-day growth, cell detachment was performed with 1 × Trypsin, and 5 ml corresponding fresh medium without FBS was added into each T25 flasks to suspend the cells. Cell counting was conducted on Beckman Vi-CELL XR Cell Viability Analyzer. The medium screening procedure of Vero cells was same as that of HEK293T cells.

### Cell subculture in T-flasks and multilayer flasks

T-Flasks were inoculated with a seeding density of 2 × 10^4^ cells/cm^2^, and grown in 37 °C with 5% CO_2_ incubator. When the cultures became confluent after 3–4 days growth, cell detachment procedures were carried out with 1 ml Trypsin/EDTA (1×) per 25 cm^2^ growth area following a D-PBS wash. Then the flasks or multilayer flasks were placed at 37 °C incubator approximately 3 min. A subculture ratio of 1:4 to 1:6 was most often applied with these cell lines in the T-Flask and multilayer flasks expansion process.

### Cell growth in spinner flasks and WAVE 25 bioreactor

Spinner flasks were placed on Micro-Stir Slow Speed Magnetic Stirrers (#W900701-F, Wheaton) in 37 °C with 5% CO_2_ incubator. Different stir speeds, 60 rpm (Spinner 125, 500 ml), 55 rpm (Spinner 1000 ml), and 50 rpm (Spinner 3000 ml), were set for mixing. Microcarriers Cytodex-1 of 3 g/l were used in all spinners. The spinners were inoculated with a seeding density of 2 ± 0.5 × 10^5^ cells/ml (Rourou et al. 2009). After 2 or 3 days, medium exchanges (30% of total volume) were performed for nutrient replenishment and cell confluence was achieved on day 3 or day 4.

For scale-up process study of Vero cells, WAVE 25 bioreactor as seed train to replace 3 units of spinner 3000 was also investigated (Genzel et al. 2006; Kumar and Starly 2015). The rocking speed setting and rocking motion were very critical for WAVE 25 microcarriers culture. A rocking speed of 12 rpm and a 6° angle was set in the first 2 days post inoculation. The speed was increased to 15 rpm in the following culture days. The rocking motion was set to 100% for a more smoothly rock (Genzel et al. 2006).

Cell morphology and attachment status to microcarriers were evaluated using an inverted microscope (Olympus CKX41, CKX53). Cell density was determined by crystal violet-citric acid method (Kurokawa and Sato 2011) and/or automated cell counter NucleoCounter^®^ NC-200™.

### Cell trypsinization on microcarriers and bead-to-bead transfer

When cell confluence was achieved on microcarriers in spinner, the microcarriers were settled by stopping agitation and the supernatant was decanted off carefully in biosafety cabinet (BSC). Microcarriers were then rinsed twice using D-PBS and once with DPBS-1 × EDTA of 30% initial culture volume (iCV) per time. Then 15% iCV of 1 × Trypsin (for HEK293T cells) or 8% iCV of 2 × Trypsin (for Vero cells) were added into the microcarriers. The cell trypsinization process was performed in 37 °C Shaking Water Baths (GLS Aqua 12 Plus, Grant) with gentle mixing every 2 min. After 15–30 min, more than 90% cells were detached from the microcarriers. The microcarriers were settled 2–3 min and the cell supernatants were pumped into a transfer bottle. Washing the microcarriers 2 or 3 times using 20% iCV of DMEM media to elevate cell recovery. Then cell counting was conducted and the cells were prepared to inoculate a new culture. A subcultivation ratio of 1:4 is recommended in microcarrier bead-to-bead expansion processes.

### Cell growth comparison on fresh microcarriers and previously populated microcarriers

One of two inoculation methods are typically applied following cell trypsinization as part of the microcarriers scale up process. One method is separating cells from previously populated (spent) beads by mesh or settling microcarriers and only transferring cells to next culture vessel. The advantage of this method is that the cells are evenly distributed across all microcarriers. However, this method will lose some cells, as it is difficult to get 100% of the cells. This will result in decrease in the potential expansion ratio in scale up process. The other method is transferring all the cells and spent beads mixture to next culture vessel following trypsinization. This method has the highest cell utilization and can simplify the operation. However, this may result in uneven cells distribution and differential growth rates on new microcarriers and spent microcarriers.

In this study, two 500 ml spinner flasks were inoculated with Vero cells at a target initial density of 2 × 10^5^ cells/ml. For one spinner, all fresh microcarriers were used and only seed cells (without spent beads) were transferred into the spinner; but for another spinner, cells and partially spent microcarriers (just detached the cells from them) were transferred in. The two spinners were placed in 37 °C with 5% CO_2_ incubator. Daily samples were taken to observe cell morphology on microcarriers and determine cell density. The microcarriers were settled and 30% of spent media volume was exchanged with fresh media on day 3 and day 4, respectively.

### Microcarrier scale-up culture studies in single-use stirred tank bioreactor

#### Determination of minimum stirring speed for a homogeneous mixing

Microcarriers maybe settle or distribute inhomogeneous because of insufficient mixing at low agitation speed. Uniform suspension and distribution of microcarriers is very important. It not only provides more surface area for cells attachment, but also avoids microenvironments resulting in nutrients and/or dissolved oxygen (DO) depletion. In this study, 10, 15, 20, 25, 30, 35, 40, 45 rpm were set for microcarriers mixing studies in XDR-50 system with culture volume of 20 l to define the minimal agitation speed. Samples were taken out after a minimum of 20 min at each of agitation speeds. 2 ml mixture was added to a Moisture Analyzer (HG63-P, Mettler-Toledo), and the content of microcarriers was detected after 5 min drying to constant weight. Once the mixing speed is sufficient, the microcarriers achieved uniform mixing and this speed is identified as the minimum stirring speed.

### Determination of a suitable agitation speed for bioreactor cell culture

A key parameter in bioreactor scale up process is agitation speed. This can be calculated by tip speed or power input per volume. Insufficient agitation speed will result in low mixing efficiency and uneven distribution of cells and nutrients in the bioreactor. However excessively high agitation speed can shear force damage or stress to cells and impact cell growth and production. When choosing an agitation speed for scale up, in addition to the minimum stirring speed, the following two parameters were also considered (Connon 2017; Merten 2015):

#### Power input per volume

1 $$ {\text{P}}/{\text{V }} = {\text{ N}}_{\text{p}} {\text{n}}^{ 3} \uprho {\text{ d}}^{ 5} /{\text{V}} $$

Here P is power input (W/m^3^), V is volume (m^3^), N_p_ is impeller’s Newton number or impeller power number, n is agitation speed (r/s), ρ is culture medium density (kg/m^3^), d is impeller diameter (m).

#### Kolmogorov eddy size

2 $$ \upeta  =  \left[ {\upnu^{ 3} /\left( {{\text{N}}_{\text{p}} {\text{d}}^{ 5} {\text{n}}^{ 3} /{\text{V}}} \right)} \right]^{ 1/ 4} $$

where η is Kolmogorov eddy size (m), ѵ is kinematic viscosity (m^2^/s), other symbol’s meanings are same to Eq. (1).

### Determination of aeration rate

Another important parameter for cell growth and scale up in stirred tank bioreactors is the aeration rate, which includes the flow rate or sparger rate for compressed air, oxygen, and carbon dioxide. Compressed air and oxygen provide dissolved oxygen for cell growth, and compressed air can also function to regulate culture pCO_2_ below inhibitory or suboptimal levels. In microcarrier culture processes, high aeration rates can result in significant stress or damage to cells, thereby impacting attachment and growth. This is especially true for the cell types that are not firmly attached to microcarriers, such as HEK293T cells. In order to decrease aeration rate, the mixture aeration (1:4) of compressed air and oxygen were cascaded to DO control in XDR-50 and XDR-200 culture processes. Carbon dioxide is used to adjusted pH value in culture process, however too high CO_2_ gas flow rate also affect the cell attachment in the microcarrier. In order to reduce the influence of CO_2_ ventilation on cell attachment in the early stage of cell inoculation, pH value was adjusted to the low limit of pH control, and the CO_2_ gas flow rate was capped at 0.25 l/min.

### Microcarrier scale-up in XDR-50 and XDR-200 bioreactors with HEK293T and Vero cells

**Fig. 1:**
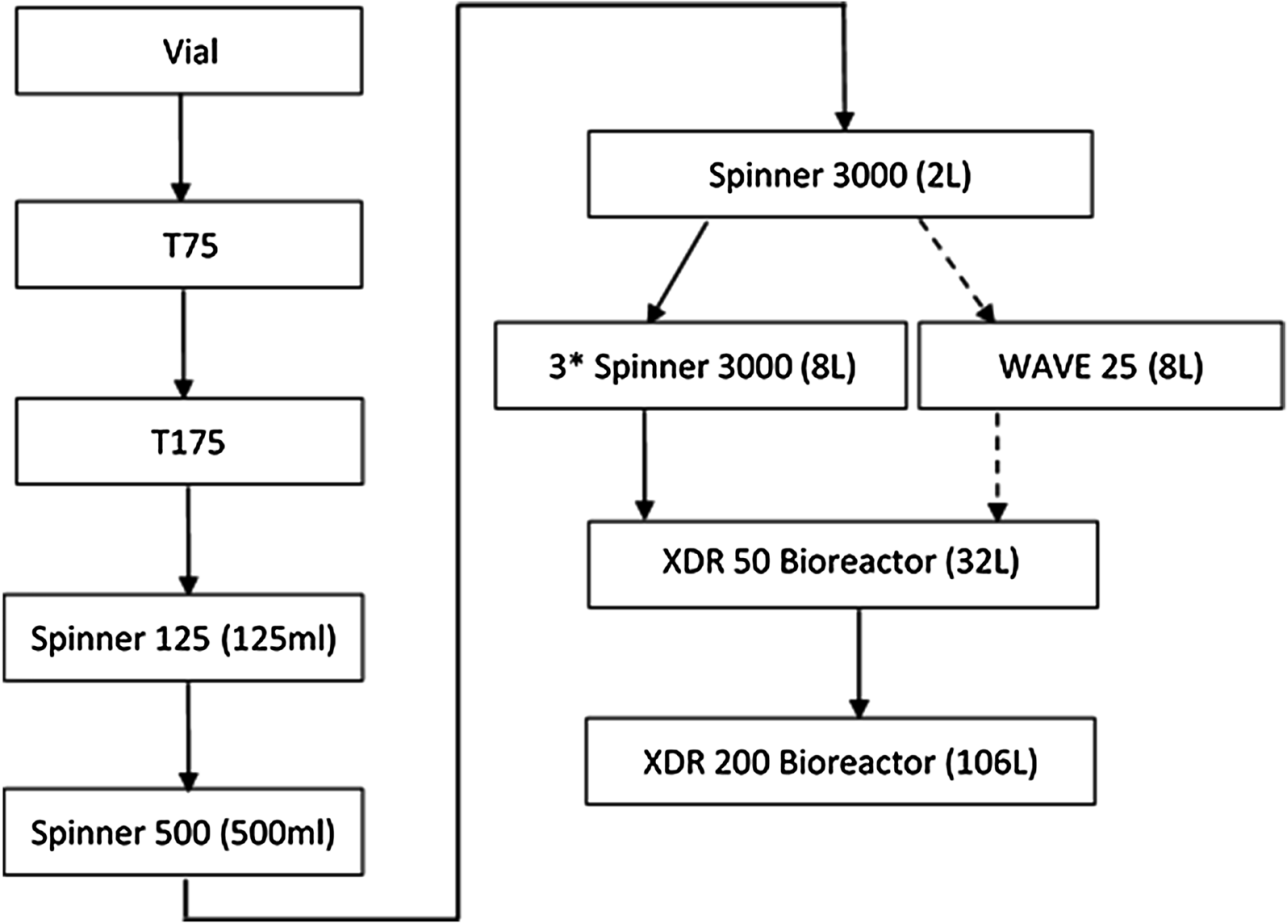
Expansion flow chart of HEK293T cells and Vero cells. The dotted arrow represented an option to large-volume seed culture

HEK293T cells were thawed into T75 flask and subcultivated to T175, then expanded in 125 ml to 3000 ml spinner flasks. The cells from (3) 3000 ml spinner flasks were used to inoculate an XDR-50 bioreactor (Fig. 1). The initial culture volume was 32 l with the inoculum density of 2 × 10^5^ cells/ml and 3 g/l of Cytodex-1 microcarriers. The XDR-50 bioreactor was set to a temperature of 37 °C, and the agitation rate was set to 40 rpm with down pumping mode. Continuous agitation was applied during cell attachment phase and cell growth phase. The pH was maintained at 7.10 ± 0.1 through CO_2_ sparger and 7.5% NaHCO_3_ auto pump. DO was maintained at 40 ± 10% of air saturation by cascading air sparger and oxygen sparger. Samples were taken daily from XDR-50 bioreactor to examine cell morphology and concentration, and the metabolites were also analyzed by Nova Profile 400. Glucose concentration was selected as an indicator for media exchange rate. The culture was stopped once cell confluence was achieved on all microcarriers.

Vero cells expansion paradigm was similar to HEK293T cells using the XDR-50 bioreactor. Specifically, 3 g/l Cytodex-1 microcarriers and 32 l culture volume were applied for Vero cell growth in XDR-50 bioreactor, with the initial inoculum of 2 × 10^5^ cells/ml. The agitation rate was set at 40 rpm, which correlates to 6 W/m^3^ of power input per volume. Other key bioreactor process parameters were set to 37 °C, pH 7.10, DO 40%, and the medium exchange strategies were the same as the HEK293T cell cultivation in XDR-50. The cells were detached by 2 × Trypsin solution in a harvest container once the microcarriers were confluent, then bead-to-bead transfer process was conducted in order to scale to the XDR-200 bioreactor. The Vero cells inoculum and Cytodex-1 microcarrier concentration in XDR-200 bioreactor were similar to the XDR-50, and the culture volume was 106 l. The agitation speed was set to 60 rpm which had a same power input per volume of 6 W/m^3^, and the eddy size was controlled at 90–95 μm. Oxygen and carbon dioxide sparger gas flow rates were cascaded to DO (40% ± 10%) and pH (7.00 ± 0.20) control, respectively. The maximum sparger flow rate was capped at 1 l/min. The feeding strategy and cell counting was same to XDR-50 and the culture was stopped once the confluence achieved on all microcarriers.

### Statistical analysis

Data in this work was analyzed by Microcal Origin 6.0 and presented as mean ± SD. Comparison was performed by Student’s t-test or One-Way ANOVA analysis. *P* value < 0.05 was considered statistically significant.

## Results

### Cell culture medium screening for HEK293T cells

Basal medium and serum play a critical role in reaching high cell density and sustaining long-term cell growth. In this study, media screening experiments for cell growth of HEK293T cells and Vero cells were conducted as a first step. Three groups of media, naming as (I) MEM + FBS, (II) M199 + FBS, (III) DMEM + FBS, were employed for each kind of cell.

**Fig. 2:**
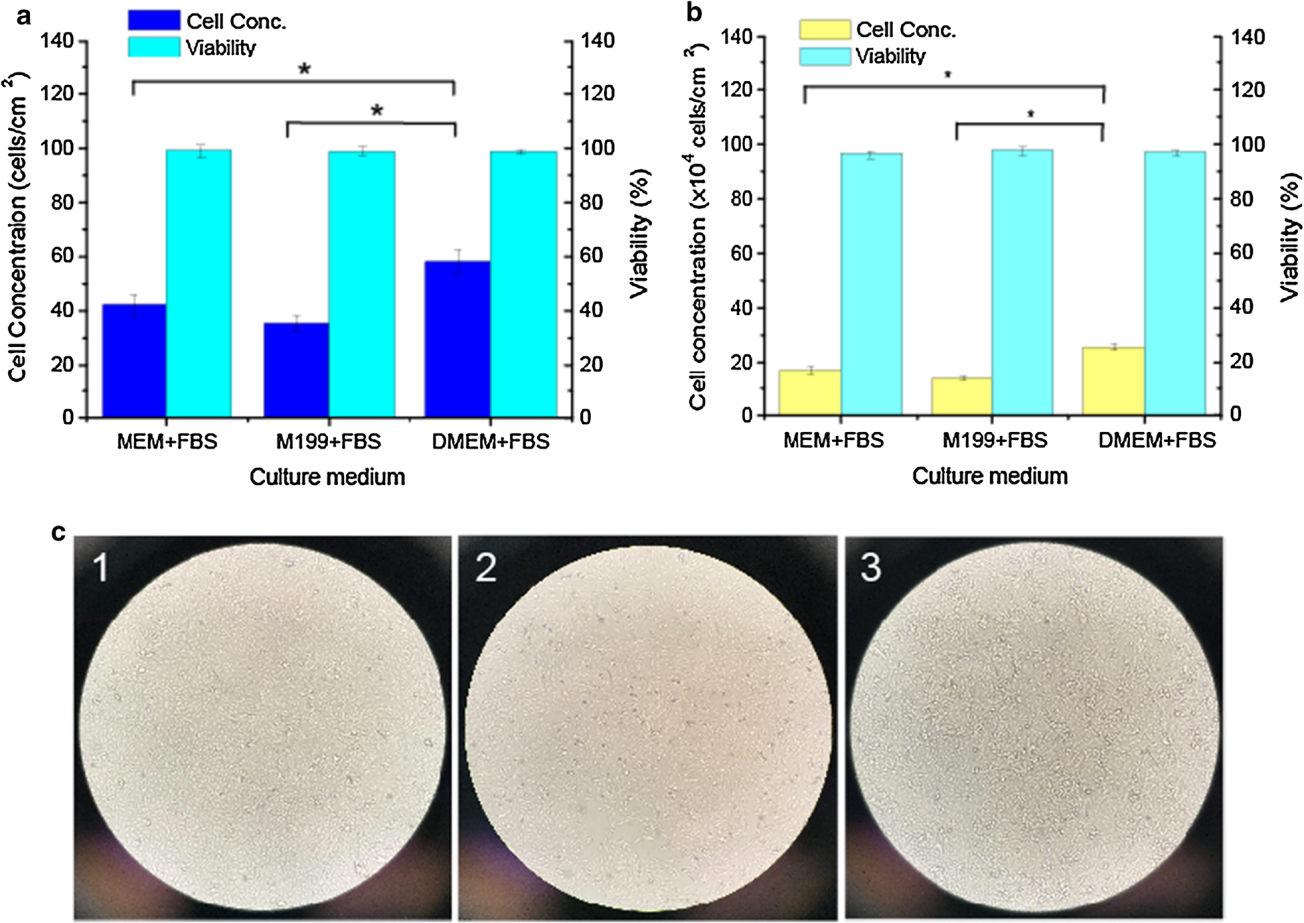
Cell culture media screening results. **a** Comparison of HEK293T cell growth in different culture media (n = 5). **b** Comparison of Vero cell growth in different culture media (n = 5). **c** Vero cell growth pictures in different media on day 4, (1) MEM + FBS, (2) M199 + FBS, (3) DMEM + FBS

For HEK293T cells, the initial cell inoculum concentration was 2 × 10^4^ cells/cm^2^ in each group media. After 4 days growth, the best cell growth performance with an average 58.1 ± 4.6 × 10^4^ cells/cm^2^ was achieved in the DMEM + FBS group, compared to the MEM + FBS group (42.3 ± 3.9 × 10^4^ cells/cm^2^) and the M199 + FBS group (35.4 ± 2.9 × 10^4^ cells/cm^2^) (Fig. 2a). The specific growth rate and doubling time were 0.84/day and 20 h in the DMEM + FBS group. This was followed by the MEM + FBS media group (0.76/day and 21.9 h) and M199 + FBS (0.72/day and 23.1 h). In the process, the cell viability was also determined, however no significant difference was observed with viabilities of 98.7% to 99.1% in the three kinds of group media. For Vero cells, the highest cell concentration was reached to 25.6 ± 1.1 × 10^4^ cells/cm^2^ in the DMEM + FBS group, which increased 50.1% compared to the MEM + FBS group and 89.6% of the M199 + FBS group (Fig. 2b). In addition, more globular dead cells were found in the M199 + FBS media group (Fig. 2c).

Altogether, DMEM + FBS media group were more suitable for HEK293T cells and Vero cells growth, and ultimately selected for subsequent bead-to-bead transfer studies and scale-up process studies.

### HEK293T cells and Vero cells bead-to-bead transfer studies in spinner flasks

**Fig. 3:**
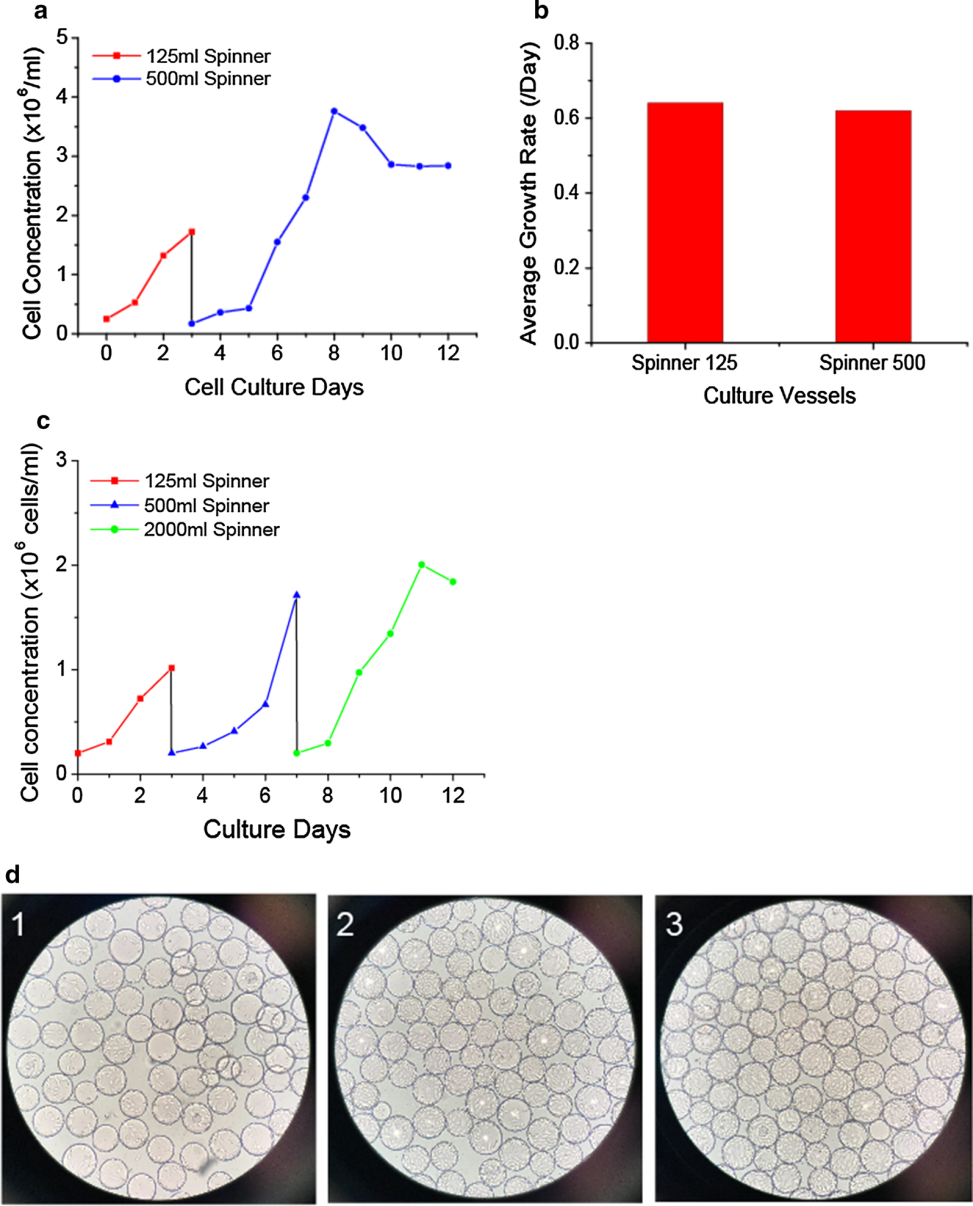
Bead-to-bead transfer studies in spinner flasks. **a** HEK293T cell growth curve of bead-to-bead transfer study in 125 ml and 500 ml spinner flasks. **b** Growth rate comparison of HEK293T cells before and after microcarrier bead-to-bead transfer. **c** Vero cells bead-to-bead transfer studies in 125, 500, and 3000 ml spinner flasks. **d** Vero cell growth pictures in 3000 ml spinner flask on day 1, day 3 and day 5

HEK293T cells and Vero cells with 3 g/l of Cytodex-1 microcarriers were cultured in spinner flasks which were placed on Micro-Stir Slow Speed Magnetic Stirrers at 37 °C and 5% CO_2_ incubator. Continuous stirring regime was employed in all spinners culture process. The cell attachment rates to microcarriers were determined by checking free cells disappearance from the media. After inoculation 4 h, the cell attachment rate was higher than 95% in all spinner cultures, and percentage of unoccupied beads was extremely low. The cell distribution and morphology were checked by inverted microscope. For HEK293T cell culture, each microcarrier became confluent after 3–5 days growth. The peak cell concentration reached 3.5 × 10^6^ cells/ml with an average cell growth rate of 0.64/day in 125 ml spinner flask and 0.62/day in 500 ml spinner flask (Fig. 3a). There was not a significant difference before and after microcarrier bead-to-bead transfer process (Fig. 3b). For Vero cells culture, the peak cell concentration achieved more than 2 × 10^6^ cells/ml with an average growth rates of 0.44–0.59/day (Fig. 3c). No microcarrier aggregates were found during the culture processes (Fig. 3d).

Collectively, these results showed that Vero cells and HEK293T cells combining with microcarriers can grow very well in spinner flasks, and the microcarrier bead-to-bead transfer processes have been setup.

### Effects of fresh microcarriers and previously populated microcarriers on cell growth

**Fig. 4:**
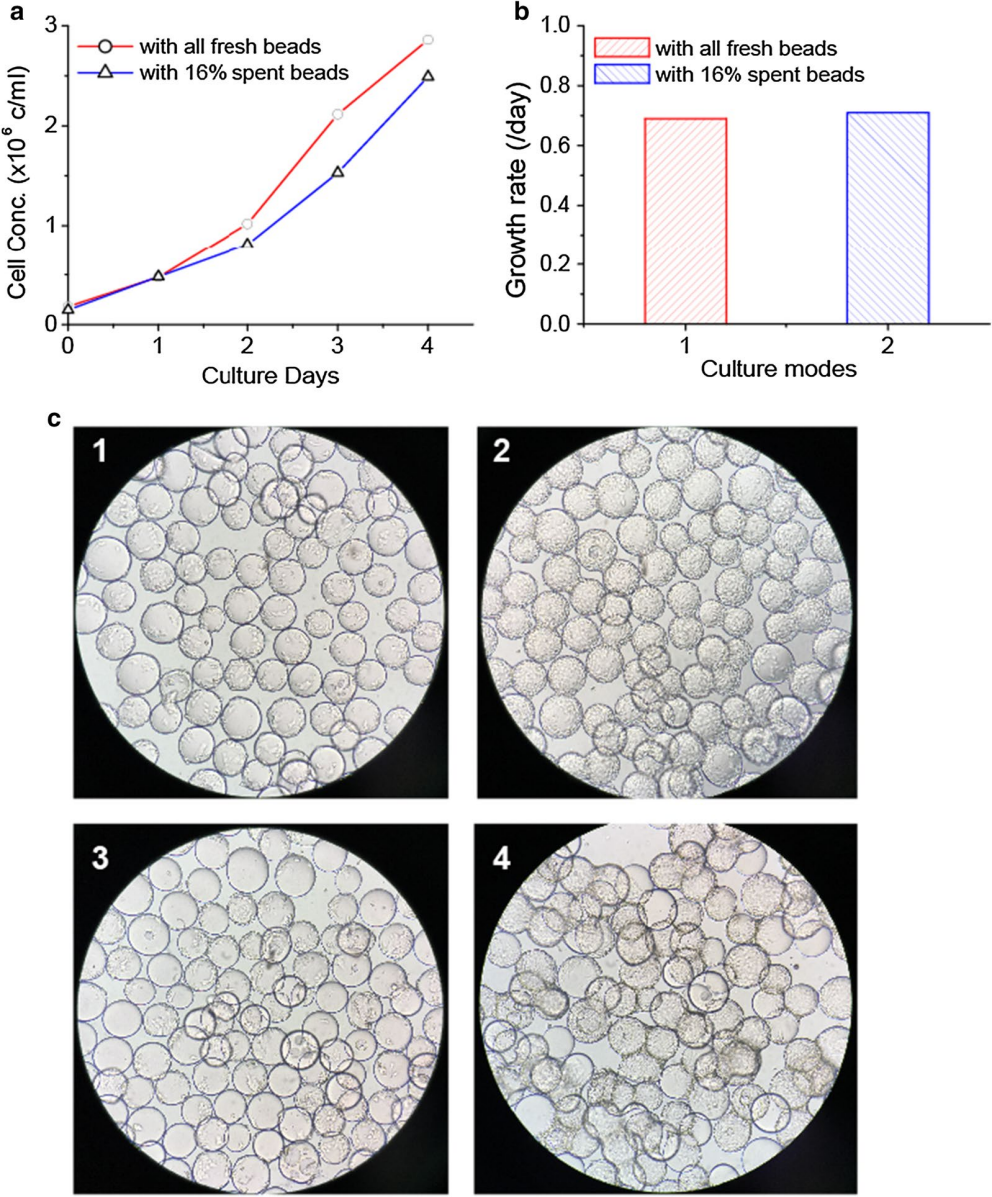
Effects of fresh microcarriers and partial spent microcarriers on cell growth. **a** Vero cell growth curves in all fresh microcarriers and partial spent microcarriers. **b** Vero cell growth rate comparison in previously described conditions above. **c** HEK293T cell growth pictures. Images 1 and 2 (above) are day 1 and day 3 of HEK293T cell growth on all fresh microcarriers. Images 3, 4 (above) are day 1 and day 3 of HEK293T cell growth on partial spent microcarriers

Cell growth comparison on all fresh microcarriers and previously populated (spent) microcarriers was investigated in this study. For Vero cell cultures, the peak cell concentration reached 2.86 × 10^6^ cells/ml on all fresh microcarriers on day 4, which was slightly higher than that of spent microcarriers (2.49 × 10^6^ cells/ml) (Fig. 4a). However, there was no significant difference in cells distribution between fresh microcarriers spinner and partial spent microcarriers spinner (pictures not shown). Meanwhile both the growth rates reached 0.69–0.71/day and didn’t show a distinct difference between those two culture modes (Fig. 4b). For HEK293T cell cultures, the cells achieved confluence, and cell distributions were homogeneous in the spinner cultures containing all fresh microcarriers. However, in the spinner of containing partial spent microcarriers, we found that some microcarriers were not fully populated, and more bead aggregates were formed. Furthermore, the aggregate size (3–5 beads sticking together) was bigger than that of the fresh microcarriers spinner (2–3 beads) (Fig. 4c).

Taken together, these results indicated that the spent microcarriers and digested cells mixture can be transferred to next step vessel for further expansion for Vero cell culture processes; but it is more recommended that the digested cells and spent microcarriers should be isolated, and only the cells are transferred to next vessel for HEK293T cell expansion processes.

### HEK293T cell and Vero cell cultures in WAVE25 bioreactors

**Fig. 5:**
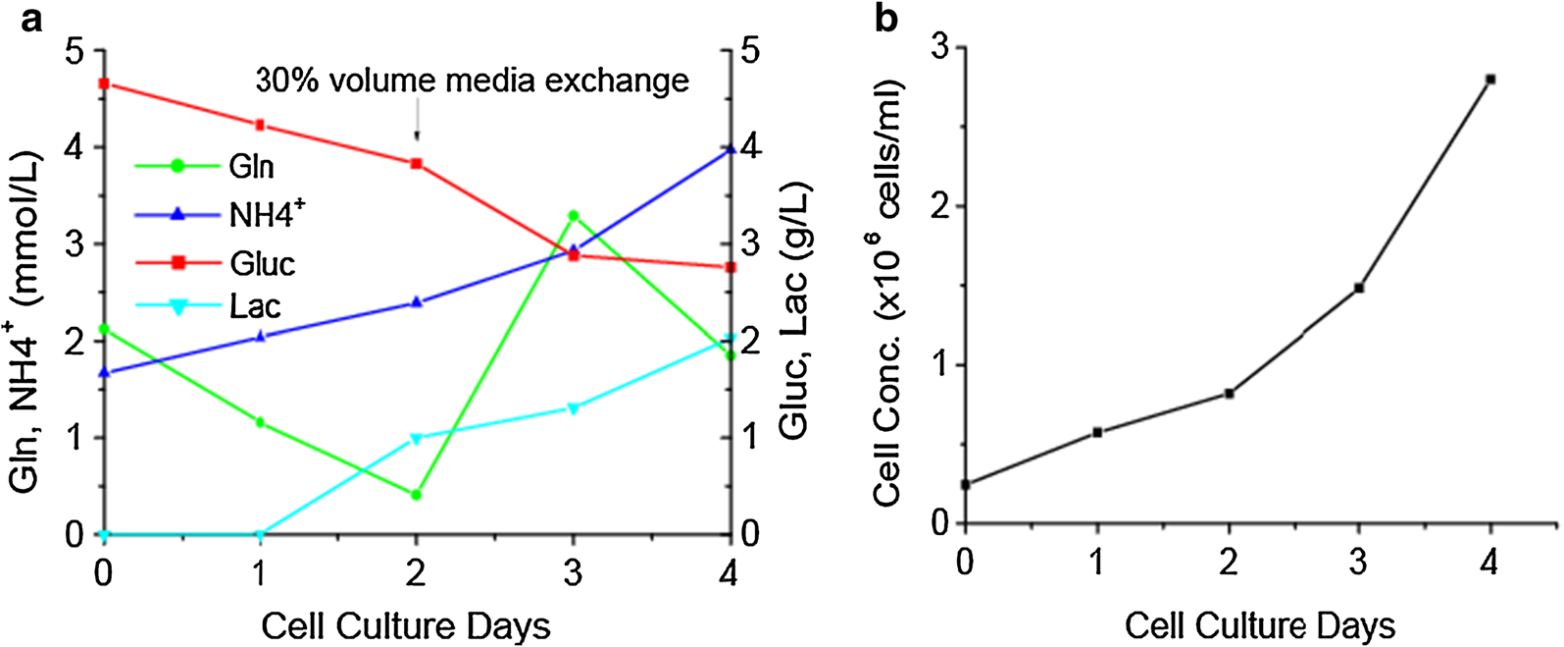
Vero cell growth in WAVE25 bioreactor. **a** Metabolite analysis of Vero cell growth in WAVE25 (on day 2, 30% CV media exchanged, and additional 20 mM glutamine was supplied in the bioreactor). **b** Vero cell growth curve in WAVE25

To investigate the feasibility of using the WAVE bioreactor as a seed vessel for adherent cell expansion, Vero cell and HEK293T cell cultures were grown in the WAVE25 bioreactor. For Vero cell culture, the inoculum cell concentration was 0.245 × 10^6^ cells/ml. In the culture process, the metabolite concentrations were checked daily to maintain glucose concentration higher than 1 g/l and glutamine concentration greater than 1 mM. To avoid nutrient depletion, 30% culture volume of media was exchanged using fresh media on day 2, in addition, 100 ml of 200 mM glutamine was added into the bioreactor to increase the total glutamine concentration to 4 mM (Fig. 5a). The peak cell concentration reached 2.8 × 10^6^ cells/ml on day 4 (Fig. 5b) and the average growth rate was 0.61/day. The Vero cell morphology indicated the culture was very healthy and the cell distribution on all microcarriers was homogeneous. For HEK293T cells cultured in WAVE25 bioreactors, even though different culture parameters were evaluated, the seed cells didn’t attach to the microcarriers. The cells aggregated, resulting in many cell-aggregates similar to globules (Pictures not shown).

These results indicated that WAVE25 can be used as a culture vessel or seed train for Vero cells expansion process; but is not suitable for HEK293T growth or expansion.

### Microcarrier scale-up culture studies in single-use stirred tank bioreactor

#### Determinate suitable agitation speed for scale up in different bioreactors

**Fig. 6:**
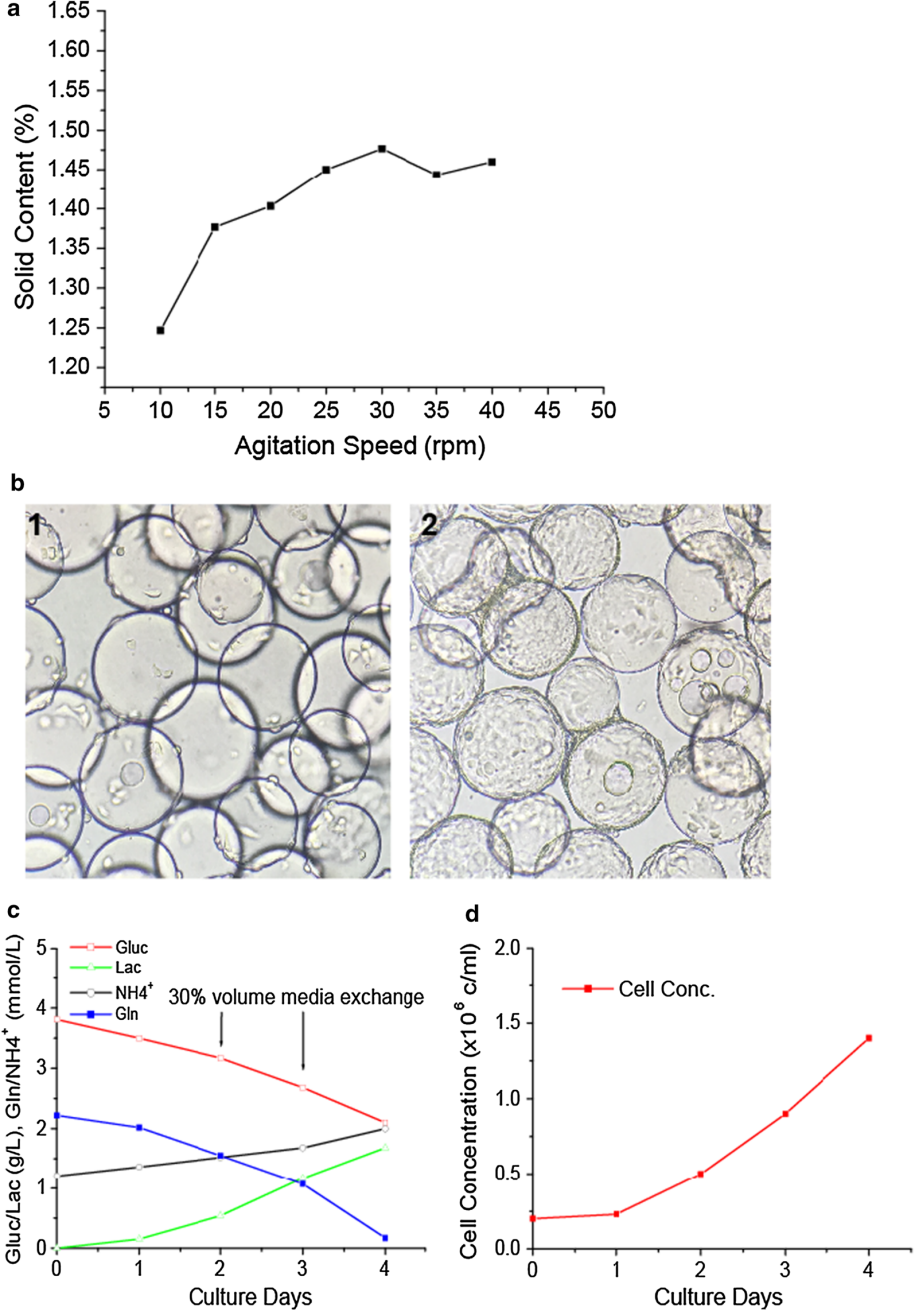
HEK293T growth in XDR-50 bioreactor. **a** Minimum stirring speed determination of XDR50 for microcarrier homogeneous mixing. **b** HEK293T cell growth pictures on day 0 4 h and day 4. Some microcarrier aggregates can be found on day 4. **c** Glucose, lactate, and glutamine, NH_4_^+^ profiles of HEK293T cells grown in XDR50. **d** HEK293T cells growth concentration curve in XDR-50 bioreactor

Microcarriers may settle if an insufficient agitation speed is applied, which will lead to non-homogeneous microcarrier distribution in bioreactor. In this study, to define the minimum agitation speed, 10, 15, 20, 25, 30, 35, 40, 45 rpm were set for microcarriers mixing studies in XDR-50 system with a volume of 20 l. The graph below (Fig. 6a), shows the solid content percentage is maintained at a relatively constant value once the stirring speed exceeds 25 rpm. Therefore, 25 rpm was determined as the minimum stirring speed for the following microcarrier culture in XDR bioreactor.

In this study, 40 rpm was used for Vero cell and microcarrier culture in XDR-50. The stirring speed correlates to 6 W/m^3^ of power input per volume and the cells grew very well. When the culture was scaled up to XDR-200 bioreactor, the key parameter of power input per volume was kept equivalent. For the agitation speed, 60 rpm for XDR-200 process was derived from the Eq. (1) with a power input per volume of 6 W/m^3^, similar with XDR-50 bioreactor.

Another key parameter, Kolmogorov eddy size, was also considered to confirm whether the agitation speed (60 rpm) is appropriate in the scale-up process. The eddy size was significantly affected by the agitation rate and was calculated using the Eq. (2). In order to reduce the damage of cell and microcarrier caused by fluid shear stress, Eddy size should avoid being similar to cell diameter and microcarrier diameter. In XDR-200 bioreactors, the eddy sizes were 90–95 μm when 60 rpm was applied. They were significantly smaller than microcarrier’s size (190 μm) and bigger than Vero cells size (15–20 μm). The fluid perturbations shouldn’t damage the microcarriers and cells. So, 60 rpm was finally determined as the agitation speed for XDR-200 expansion process.

### HEK293T cells cultivation in XDR-50 bioreactor

The XDR-50 bioreactor was inoculated with a cell concentration of 2 × 10^5^ cells/ml. More than 95% of cells attached to microcarriers after inoculation 4 h, and the cells achieved confluence on most of microcarriers after 4 days growth (Fig. 6b). 30% volume (10 l) of spent media was exchanged with fresh medium to prevent nutrient limitation on day 2 and day 3. The glucose concentration was maintained higher than 2 g/l throughout the culture process. Meanwhile, some metabolic waste products were removed from the culture by the medium exchange. The lactate was kept at lower than 2 g/l, and the ammonia was kept at lower than 2 mM. The profiles of nutrients, and metabolic waste products were given (Fig. 6c). With the cell growth, some small microcarrier aggregates, which were composed of 2 or 3 microcarriers, were found on day 3 and day 4. The peak cell concentration was achieved at 1.5 × 10^6^ cells/ml on day 4, and the cell growth profile was shown in Fig. 6d.

These data showed that HEK293T cells combining with microcarriers can grow well in XDR-50 bioreactor.

### Vero cell cultivation in XDR-50 and XDR-200 bioreactors

**Fig. 7:**
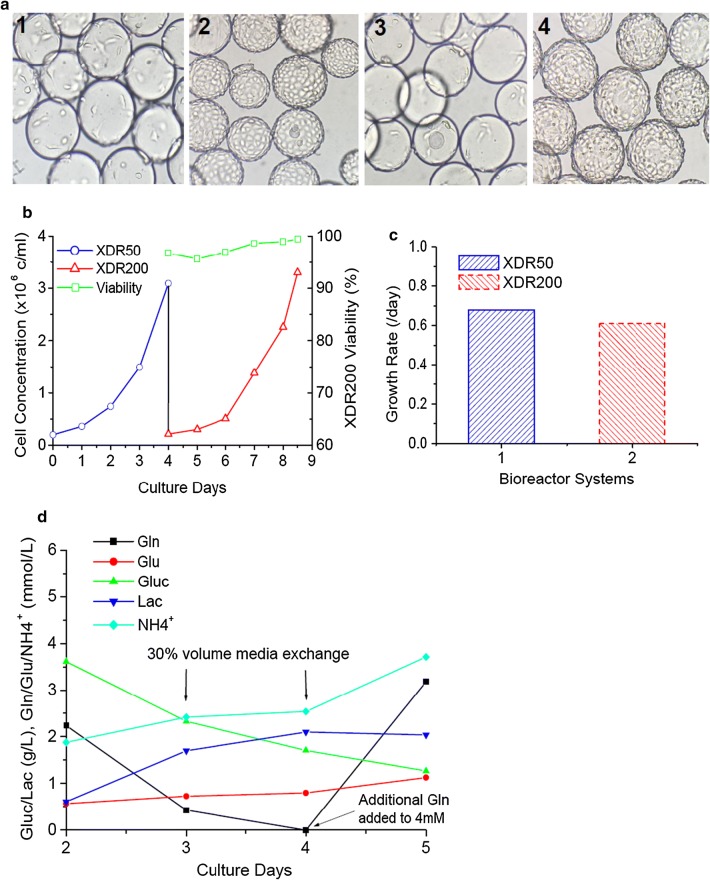
Vero cells growth in XDR-50 and XDR-200 bioreactors. **a** The pictures of Vero cell growth in XDR-50 on day 1 (1) and day 4 (2), and in XDR-200 on day 1 (3) and day 4.5 (4). **b** Cell growth concentration curve in XDR50 and XDR200 bioreactors. **c** The average specific growth rates in XDR-50 and XDR-200 bioreactors. **d** Cell metabolite curves in XDR200

8 l of Vero cells and microcarriers from (3) 3 l-Spinner flasks were detached using a 2 × Trypsin solution and expanded into XDR-50 bioreactor with a split ratio of 1:4. The initial inoculum was 2 × 10^5^ cells/ml with a 32 l culture volume. After greater than 8 h, all the cells attached to microcarriers and spread for growth. The cells achieved confluence on day 4 in XDR-50 bioreactor (Fig. 7a). Bead-to-bead transfer was carried out and the culture was scaled up to XDR-200 bioreactor with a culture volume of 106 l. The cells achieved confluence after 4.5 days growth, with a homogenous distribution on all microcarriers in XDR-200 bioreactors (Fig. 7a). To avoid the depletion of nutrients, 30% culture volume (10 l for XDR50, and 32 l for XDR200) of spent media was exchanged with fresh medium on day 2 and day 3. During the culture process, the glucose and glutamine concentrations were maintained higher than 2 g/l and 1 mM respectively. The peak cell concentrations were achieved at 3.1 × 10^6^ cells/ml and 3.3 × 10^6^ cells/ml in XDR-50 and XDR-200 bioreactors at day 4 and day 4.5, respectively (Fig. 7b). The average specific growth rates were consistent at 0.68/day and 0.61/day in XDR-50 and XDR-200 bioreactors (Fig. 7c).

The results showed that the Vero cell expansion paths were feasible, and the cells combining with microcarriers can grow well in XDR-50 and XDR-200 bioreactors.

## Discussion

For gene therapy and cell therapy, virus production in multiple lawyer flasks has been reported by Levine et al. (2017), which supported the launch of the first gene therapy drug (Tisagenlecleucel). Using this production mode, maybe the virus yields meet the demand when the project is in clinical phase or just goes into market. However, as the number of patients receiving gene therapy increases every year, the production of a large quantities of viruses will become a limiting factor (Maartens et al. 2017). The objective of the present work was to develop microcarriers bead-to-bead transfer processes and establish the scale-up cultivation processes for HEK293T cells and Vero cells in 50–200 l single-use XDR bioreactors. The ultimate goal is to develop virus large-scale production process to meet the need of gene therapy and vaccine industry.

Three groups of media, including (I) MEM + FBS, (II) M199 + FBS, (III) DMEM + FBS, were compared to determine the best medium for cell growth. Cell concentration and growth rate were selected as the key evaluation criterion. For both cell line of HEK293T and Vero, the best cell growth performances were achieved in DMEM + FBS media group, with cell concentration increased more than 30%. This may be the reason that DMEM medium had higher concentration of nutrients, such as glucose, amino acid and vitamins, compared to MEM and M199 media. DMEM + 10% FBS was ultimately selected to be used for subsequent bead-to-bead transfer studies and scale-up process studies for HEK293T cells and Vero cells.

HEK293T cells and Vero cells microcarrier cultures and bead-to-bead transfer process developments were studied in different scale spinner flasks. An agitation rate of 50–60 rpm was applied to provide sufficient mixing force, resulting in the homogeneous distribution of microcarriers in the entire culture systems. A suitable inoculum concentration is especially important during inoculation phase. This helps decrease the number of empty beads, ensure an appropriate distribution of cells on each of microcarriers, and maximizes the total surface for cell growth (Ng et al. 1996). For the experiments in this study, all the cultures in spinners and bioreactors, the inoculation densities were 1.5–2.5 × 10^5^ cells/ml with 3 g/l of Cytodex-1 microcarriers, which was corresponding to a cell/bead ratio of 7–15. Using this inoculation concentration and continuous stirring regime, more than 95% cells attached the microcarriers after 4 h from the time of inoculation and the cells were distributed uniformly on all microcarriers. Furthermore, no significant lag growth phase was observed.

Microcarriers bead-to-bead transfer is a critical step for scale up culture. The feasibility of directly bead-to-bead transfer between fresh microcarriers and confluent microcarriers through bridging mode has been reported (Wang and Ouyang 1999). However, the problem of asynchrony still limits the application of this method in industry. New added microcarriers needs more days to achieve confluence, but the cells on confluent beads maybe have been out of exponential growth stage or in stationary stage. In this study, 1 × Trypsin and 2 × Trypsin solutions were used to detach HEK293T cells and Vero cells from microcarriers, respectively. The cell recovery rates were higher than 90%, and cell viabilities were higher than 95%. The cells were inoculated to a subsequent culture for scaleup with a split ratio of 1:4 to 1:6. The growth rate or doubling time didn’t show significant differences before and after microcarrier bead-to-bead transfer.

Microcarrier culture is quite different in WAVE25 bioreactor compared to agitated spinners/bioreactors, especially for some cells which are not tightly attached on microcarriers, such as HEK293T cells. In WAVE25 bioreactor, cellbag rocking motion at a user-determined speed and angle brings waves for liquid mixing and mass transfer. The cells are moving in different directions, which results in more opportunities to collide, and potentially stick together to form cell aggregates. For HEK293T cell culture in WAVE25 bioreactor, many cell aggregates were observed and less cells were attached the microcarriers. This suggests that cell aggregate formation has a direct effect on cell-bead attachment. The HEK293T cells are more likely to form cell aggregates rather than attach the microcarriers in WAVE25 bioreactor. However, HEK293T cells had a good cell-bead attachment in stirred spinner flasks and bioreactors with a fast cell attachment and even distribution on microcarriers. For Vero cells culture in WAVE25 bioreactor, the cells had a good cell attachment and distribution on microcarriers with rocking speed of 12–15 rpm and angle of 6°, which can be attributed to the larger cell-bead attachment rate than cell aggregate formation.

In XDR-50 bioreactor combining 3 g/l of Cytodex-1 microcarriers and agitation speeds of 40 rpm were applied for HEK293T and Vero cells. When the scale-up culture process was conducted, 60 rpm was determined as the agitation rate for Vero cell growth in XDR-200 bioreactor. This corresponds to a power input per volume of 6 W/m^3^, similar to that of XDR-50 bioreactor. In addition to power input per volume, eddy size was also considered during the determination of the agitation speed. The eddy size was maintained at 90–95 μm to avoid shear force damaging the cells and microcarriers. In this study, the minimum stir speed was also investigated to avoid microcarrier settling and to maintain homogenous mixing in bioreactors. 3 g/l Cytodex-1 microcarriers (surface area 4400 cm^2^/g) corresponding the attachment surface of 13.2 cm^2^/ml, Vero cells achieved the peak cell concentration at 3.1 × 10^6^ cells/ml (23.4 × 10^4^ cells/cm^2^) and 3.3 × 10^6^ cells/ml (25.4 × 10^4^ cells/cm^2^) in XDR50 and XDR200 bioreactors, respectively. The same surface area for attachment produced the similar cell numbers in T-Flask (25.6 ± 1.1 × 10^4^ cells/cm^2^) and large scale single-use bioreactor combining microcarrier culture processes, even the microcarrier bead-to-bead transfer were conducted 4–5 times in the whole scaleup processes.

In conclusion, the present study developed microcarrier bead-to-bead transfer processes of HEK293T cells and Vero cells in spinner flasks, and the transfer techniques and culture processes were successful scaled up to disposable XDR-50 bioreactor and XDR-200 bioreactors. This study can give further intuitivism to the feasibility of applying single-use bioreactors and microcarrier for large scale cell culture applications, including viral vector production in gene therapy and vaccine production.

## Data Availability

Not applicable.

## Abbreviations

Vero cells: African green monkey kidney cells
HEK293T cells: human embryonic kidneys 293T cells
XDR: Xcellerex disposable reactor
DO: dissolved oxygen
